# Supplementary material for: Spatial Variation in Stream-Water Bacterial Communities Across Sites Differing in Wild Chinese Giant Salamander Detection Frequency
Source: Biology (Basel). 2026 Sep 18;15(18):1647. doi: 10.3390/biology15181647 (PMC13604818; doi:10.3390/biology15181647)
Supplement: Supplementary file 1 [file biology-15-01647-s001.zip › Data Related to “Spatial Variation in Stream-Water Bacterial Communities across Sites Differing in Wild Chinese Giant Salamander Detection Frequency”/Microbial Data/result/08.FunctionPrediction/08.FunctionPrediction_ReadMe.pdf]

扩增子结果文件补充说明（08）

2023年 8 月 28 日

8. Funnction_prediction【功能预测】

1. Picrust2【Picrust2预测】

1.1 predicted【picrust2（COG,EC,KO,PFAM,TIGRFAM,pathway) 数据库预测结果】

1.1.1 *_metagenome_descrip.tsv【(COG,EC,KO,PFAM,TIGRFAM,pathway) 数据库预测结果】

1.1.2 *_seqtab_norm.tsv.gz【基于预测 16S 拷贝数校正的特征表, pathways_out 无此结果】

1.1.3 *_weighted_nsti.tsv.gz 【每个样本的NSTI 权重, pathways_out 无此结果】

1.2 tables 【预测结果的相对（绝对）丰度】

1.2.1 sample/group1/... 【样本及分组 预测结果的相对（绝对）丰度】

1.2.2 picrust2. {sample,group}.*.absolute.xls 【(样本,分组)各数据库预测结果的绝对丰度】

列数	列标题	含义
1	KO_Hierarchy	KO 层级
2	样本名称	各样本中各功能注释的绝对丰度值
3	KEGG_Description	功能注释信息

注：picrust2. {sample,group}.{level1,level2,level3}.absolute.xls 为KEGG（1,2,3）层级预测结果

1.2.3 picrust2. {sample,group}.*.relative.xls 【(样本，分组)各数据库预测结果的相对丰度】

1.2 barplot 【功能相对丰度柱形图，展示同物种barplot】

1.3 heatmap 【功能注释聚类热图，展示同物种heatmap】

1.4 venn 【基于功能的venn结果，展示同物种venn】

1.5 flower 【基于功能的花瓣图，展示同物种flower】

1.6 pca 【各功能 PCA 分析结果，展示同beta_pca】

1.7 ttest 【组间差异显著的功能分析各，展示同物种ttest】

2.Picrust 【Picrust预测结果】

2.1 predicted 【 KEGG 预测结果】

2.1.1 KEGG_metagenome_predictions. {biom,txt} 【 KEGG 预测结果】

2.1.2 predicted_metagenomes.KEGG_L*.txt 【 KEGG（1,2,3）层级预测结果】

2.2 tables 【预测结果的相对(绝对)丰度】

2.2.1 sample/group1/... 【样本及分组 预测结果的相对（绝对）丰度】

2.2.2 picrust. {sample,group}.*.absolute.xls 【(样本,分组)（1,2,3,k）层级预测结果的绝对丰度】

2.2.3 picrust. {sample,group}.*.relative.xls 【(样本,分组)（1,2,3,k）层级预测结果的相对丰度】

2.3 barplot【功能相对丰度柱形图，展示同物种barplot】

2.4 heatmap 【功能注释聚类热图，展示同物种heatmap】

2.5 venn 【基于功能的venn结果，展示同物种venn】

2.6 flower 【基于功能的花瓣图，展示同物种flower】

2.7 pca 【各功能PCA 分析结果，展示同beta_pca】

2.8 ttest 【组间差异显著的功能分析各，展示同物种ttest】

3. Tax4fun 【Tax4fun预测结果】

3.1 predicted 【 KEGG 预测结果】

3.1.1 KEGG_metagenome_predictions.txt 【 KEGG 预测结果】

3.1.2 predicted_metagenomes.KEGG_L*.txt 【 KEGG (1,2,3) 层级预测结果】

3.2 tables 【预测结果的相对(绝对) 丰度】

3.2.1 sample/group1/... 【样本及分组预测结果的相对(绝对) 丰度】

3.2.2 tax4fun. {sample,group}.*.absolute.xls【(样本,分组) (1,2,3,k) 层级预测结果的绝对丰度】

3.2.3 tax4fun. {sample,group}.*.relative.xls 【(样本,分组) (1,2,3,k) 层级预测结果的相对丰度】

3.3 barplot 【功能相对丰度柱形图，展示同物种barplot】

3.4 heatmap 【功能注释聚类热图，展示同物种heatmap】

3.5 venn 【基于功能的venn结果，展示同物种venn】

3.6 flower 【基于功能的花瓣图，展示同物种flower】

3.7 pca 【各功能PCA 分析结果，展示同beta_pca】

3.8 ttest 【组间差异显著的功能分析各，展示同物种ttest】

4. Bugbase 【Bugbase 预测结果】

4.1 normalized_otus 【标准化OTU 表】

4.2 otu_contributions 【表型预测结果堆叠柱形图】

4.1.1 *. {pdf,png} 【表型预测结果堆叠柱形图】

4.1.2 contributing_otus.txt 【表型预测结果表】

4.1.3 otu_table.relative.xls 【表型预测结果相对丰度表】

4.3 predicted_phenotypes 【表型预测结果比较箱线图】

4.3.1 *. {pdf,png} 【表型预测结果比较箱线图】

4.3.2 *_stats.txt 【表型预测结果箱型图绘制参数，如平均数，中位数，标准差】

4.3.3 predictions. {txt,xls} 【样本表型丰度表】

4.4 thresholds 【阈值选择表】

4.4.1 variances.txt 【不同阈值下的结果】

4.4.2 thresholds_used.txt 【表型预测分析使用的阈值】

4.4.3 *. {pdf,png} 【9表型在不同阈值下相对丰度变化】

5. funguild 【funguild预测结果】

5.1 predicted 【 funguild 预测结果】

5.1.1 funguild.sample.total.absolute.xls 【 funguild 预测结果】

5.1.2 funguild.sample.total.absolute. {guild, mode} 【 funguild (guild,mode) 预测结果】

5.2 tables 【预测结果的相对(绝对) 丰度】

5.2.1 sample/group1/... 【样本及分组预测结果的相对(绝对) 丰度】

5.2.1.1 funguild. {sample,group}.*.absolute.xls 【(样本,分组) (total,guild,mode) 预测结果的绝对丰度】

5.2.1.2 funguild. {sample,group}.*.relative.xls 【(样本,分组) (total,guild,mode) 预测结果的相对丰度】

5.3 barplot【功能相对丰度柱形图，展示同物种barplot】

5.4 heatmap 【功能注释聚类热图，展示同物种heatmap】

5.5 venn 【基于功能的venn结果，展示同物种venn】

5.6 flower 【基于功能的花瓣图，展示同物种flower】

5.7 pca 【各功能PCA 分析结果，展示同beta_pca】

5.8 ttest 【组间差异显著的功能分析各，展示同物种ttest】

6. faprotax　【faprotax预测结果】

6.1 predicted　【 funguild 预测结果】

6.1.1 faprotax.report.txt　【 faprotax 预测结果】

6.1.2 faprotax.sample.anno.absolute.xls　【 faprotax 预测结果绝对丰度】

6.2 tables 【预测结果的相对(绝对) 丰度】

6.1.1 sample/group1/...　【样本及分组预测结果的相对(绝对) 丰度】

6.1.1.1 faprotax. {sample,group}.anno.absolute.xls　【(样本,分组)预测结果的绝对丰度】

6.1.1.2 faprotax. {sample,group}.anno.relative.xls 【(样本,分组)预测结果的相对丰度】

6.3 barplot【功能相对丰度柱形图，展示同物种barplot】

6.4 heatmap　【功能注释聚类热图，展示同物种heatmap】

6.5 venn　【基于功能的venn结果，展示同物种venn】

6.6 flower 【基于功能的花瓣图，展示同物种flower】

6.7 pca　【各功能PCA 分析结果，展示同beta_pca】

6.8 ttest　【组间差异显著的功能分析各，展示同物种ttest】
